# Dietary treatment of Crohn’s disease: perceptions of families with children treated by exclusive enteral nutrition, a questionnaire survey

**DOI:** 10.1186/s12876-016-0564-7

**Published:** 2017-01-19

**Authors:** Vaios Svolos, Konstantinos Gerasimidis, Elaine Buchanan, Lee Curtis, Vikki Garrick, Jacqueline Hay, Susan Laird, Joanna Munro, Daniel R. Gaya, Richard K. Russell, Richard Hansen

**Affiliations:** 1 Human Nutrition, School of Medicine, Dentistry & Nursing, College of Medical, Veterinary and Life Sciences, University of Glasgow, Glasgow Royal Infirmary, Glasgow, UK; 2 Department of Paediatric Gastroenterology, Hepatology and Nutrition, Royal Hospital for Children, Glasgow, UK; 3 Department of Gastroenterology, Glasgow Royal Infirmary, Glasgow, UK

**Keywords:** Crohn’s disease, Exclusive enteral nutrition, Dietary therapy, Diet, Perceptions

## Abstract

**Background:**

Diet is strongly associated with the aetiology of Crohn’s Disease (CD) and exclusive enteral nutrition (EEN) is the primary induction treatment in paediatric CD. This study explored opinions around the use of EEN and alternative novel, solid food-based diets (SFDs) expressed by paediatric patients with CD, previously treated with EEN and their parents.

**Methods:**

This anonymous questionnaire surveyed families of CD patients treated with EEN over 1 year. Two questionnaire forms were completed; one asking the patients’ opinions and another referring to their main carer. This questionnaire explored participants’ demographic characteristics; acceptability of a repeat EEN course to treat a future flare (EEN repeat); their opinion on how difficult EEN would be compared to an example SFD; and their intention to participate in a future clinical trial assessing the therapeutic efficacy of an SFD in CD.

**Results:**

Forty-one families of CD patients were approached with 29 sending replies (71%). Most of our participants were positive on completing another EEN course, however the majority would choose an SFD alternative (Patients:66, Parents:72%). Both patients and their parents rated EEN to be more difficult to adhere to compared to an example SFD (*p* < 0.05), and their ratings were strongly correlated (EEN:*r* = 0.83, SFD:*r* = 0.75, *p* < 0.001). The majority of our respondents would agree to participate in a clinical trial assessing an SFD’s effectiveness (Patients:79, Parents:72%) for the management of active CD.

**Conclusions:**

While patients with CD and their families would accept an EEN repeat, the majority would prefer an SFD alternative. CD families surveyed are supportive of the development of solid food-based dietary treatments.

**Electronic supplementary material:**

The online version of this article (doi:10.1186/s12876-016-0564-7) contains supplementary material, which is available to authorized users.

## Background

Crohn’s disease (CD) is an incurable chronic inflammatory condition of the gut. It causes severe gastrointestinal and extraintestinal complications and is associated with high morbidity, poor quality of life and increased health expenditure [1].

The medical treatment for induction and maintenance of CD remission includes anti-inflammatory and immunomodulatory medication [2], whereas exclusive enteral nutrition (EEN) is established as the primary induction treatment in paediatric CD. EEN induces both mucosal and transmural healing, has up to 80% remission rates and an excellent safety profile [3]. It is however potentially restrictive and can be difficult to adhere to for long periods of time with compliance and palatability issues limiting its use especially in adult patients [4].

The strong and sustained patient interest on the role of diet in CD has been described in the literature [5]. This is also reflected by the high usage of complementary and alternative medicine among CD patients, with dietary modifications being among the most common therapies used [6]. Additionally, emerging evidence is indicating potential clinical efficacy of exclusion solid food-based diets (SFDs) [7–13].

These facts pose a pressing need and clinical demand to explore patients’ perceptions on the use of EEN and the introduction of novel SFDs for use in routine clinical practice. The aim of this questionnaire survey was therefore to report the beliefs of carers and paediatric CD patients, previously treated with EEN, on the acceptability of such dietary treatments, including within a research context.

## Methods

### Recruitment of families with CD children

An anonymous questionnaire survey was posted to all families of paediatric CD patients who had been treated with a previously described EEN protocol [14] during 2015 by the IBD team at the Royal Hospital for Children in Glasgow (RHCG). Two questionnaire forms were included: one asking the patients’ opinions and another referring to their main carer (hereafter “parent”). A stamped addressed envelope was provided for the return of the questionnaires and a reminder was sent out 2 months later to increase response rate. Both the initial posted envelope and the reminder included a cover letter explaining the reason of this questionnaire survey, but also instructing the parents and patients to complete the questionnaires separately (see Additional file 1).

### Questionnaire

A draft questionnaire was compiled by senior medical and dietetic staff who look after patients with CD. The content validity of the survey was then checked by members of the IBD team at RHCG and its readability by lay people (see Additional files 2 and 3). The survey collected information on participants’ demographic characteristics, the acceptability of an EEN repeat, and their opinion on how difficult it was to undertake an EEN course or it would be to undertake an example SFD provided to them (using visual analogue scales, translated to a scale from 1 to 100; see Q7 and Q13 of Additional file 2). It also investigated their intention to participate in a future clinical trial assessing the therapeutic efficacy of an SFD in CD. The questions asked included both open-ended and multiple-choice and we explicitly asked the participants for any further comments on their previous EEN experience or the use of an SFD. These comments were categorised as positive, negative or neutral by the investigators. The SFD was a diet template (see Additional files 2 and 3) describing an alternative exclusion diet. We ensured that no specific dietary advice was disclosed in this example template and made a specific statement on this matter.

### Recruitment of adult CD patients

Following the same approach as described above we identified adult CD patients, treated with EEN by the IBD team at the Glasgow Royal Infirmary in Glasgow.

### Statistical analysis

Categorical responses are presented with numbers and frequencies (%). Differences between ratings of the two diets by the participants were compared with 1-sample Wilcoxon signed-rank test. Correlations of parents’ and patients’ ratings were tested with Spearman’s rank correlation. Statistical analysis was performed with Minitab 16 (Minitab Ltd, Coventry, UK) and IBM SPSS Statistics 20 (IBM Corp, Armonk, NY).

## Results

Forty-one paediatric CD patients previously treated with EEN were identified and a total of 82 questionnaires were posted to them and their parents. The returned questionnaires (*n* = 58; response rate: 71%) provided information on 29 children [Median (IQR) age: 13.3 (11.1–15) years], of whom 20 (69%) were boys. The majority of them had successfully completed a course of 8 weeks on EEN (*n* = 23; 79%); 2 (7%) discontinued treatment due to lack of response and 4 (14%) due to palatability issues. Just over half of these children (*n* = 16; 55%) had to use nasogastric (NG) tube support during the treatment course (Table 1). In all 29 cases, both the child and a “parent” had completed the questionnaire, however not all questions were completed by all respondents (Table 2).

**Table 1:** Response rate, demographic characteristics and exclusive enteral nutrition experience characteristics of paediatric Crohn’s disease participants

Characteristics	*N* (%)
Response rate	29 out of 41 (71)
Parental IBD history	3 (10)
Male gender	20 (69)
Completed 8 weeks EEN	23 (79)
Experienced EEN once	22 (76)
Repeated EEN courses	7 (24)
Use of NG tube	16 (55)
Median Age (IQR)	13.3 (11.1–15.0)

*Abbreviations*: *IBD* inflammatory bowel disease, *EEN* exclusive enteral nutrition, *NG* nasogastric tube, *IQR* interquartile range

**Table 2:** Frequencies of answers by paediatric CD patients and their parents (%Yes_%No_%n/a)

Total answers *n* = 29	Treatment failed *n* = 6	Completed treatment *n* = 23	Oral consumption *n* = 13	Use of NG tube *n* = 16
If you/your child had a further flare-up of CD, do you think you/they could complete another LD course?				
P: 59_31_10 C: 59_31_10	P: 0_83_17 C: 0_83_17	P: 74_17_9 C: 74_17_9	P: 46_39_15 C: 54_39_8	P: 69_25_6 C: 63_25_13
Do you think an SFD would be better than the LD?				
P: 72_14_14 C: 66_28_7	P: 83_0_17 C: 67_17_17	P: 70_17_13 C: 65_30_4	P: 85_8_8 C: 77_23_0	P: 63_19_19 C: 56_31_13
Would you be happy to participate in such a study if doctors felt you/they needed a repeat of the LD?				
P: 79_17_4 C: 72_21_7	P: 50_33_17 C: 50_33_17	P: 87_13_0 C: 78_17_4	P: 77_23_0 C: 77_23_0	P: 81_13_6 C: 69_19_13
Would you/your child take the SFD beyond 8w if it was effective and meant less medication?				
P: 86_7_7 C: 69_14_17	P: 83_0_17 C: 67_17_17	P: 87_9_4 C: 70_13_17	P: 92_0_8 C: 85_8_8	P: 81_13_6 C: 56_19_25

Total answers; split answers based on 8w treatment completion; split answers based on the method of enteral feeds delivery

*Abbreviations*: *NG* nasogastric tube, *CD* Crohn’s disease, *LD* liquid diet, *SFD* solid food-based diet, *P* parents, *C* children, *n/a* no answer

Almost two thirds of the patients and their parents (*n* = 17; 59%) were positive on completing another EEN course in the event of a future relapse, however a higher proportion of participants thought an SFD would be better than EEN (Patients: *n* = 19; 66%, Parents: 21; 72%) (Table 2).

**Fig. 1:**
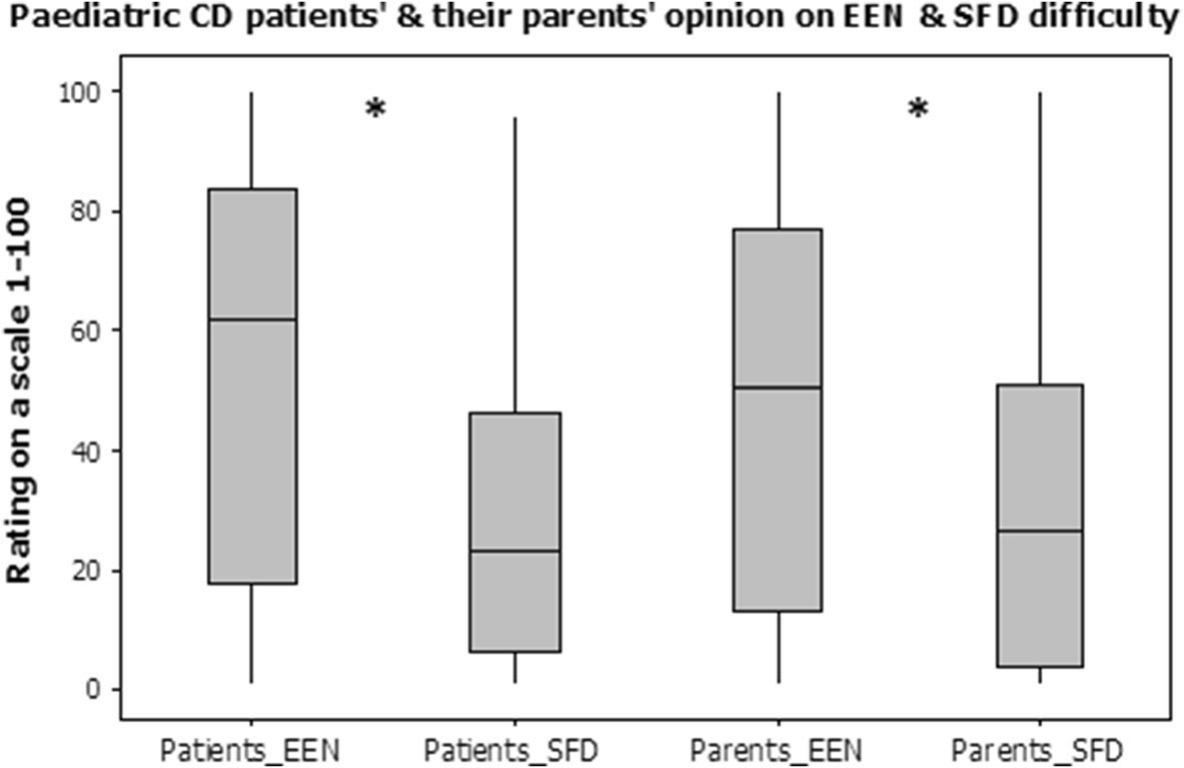
Rating of exclusive enteral nutrition and solid food-based diet difficulty by Crohn’s disease patients and their parents using visual analogue scales, translated to a scale from 1 to 100 (*p < 0.05 indicating statistically significant Wilcoxon signed-rank test between the two diets). EEN: Exclusive Enteral Nutrition, SFD: Solid Food-based Diet, CD: Crohn’s Disease

Both patients and their parents rated (on a scale from 1–100) an actual EEN course to be significantly more difficult when compared to the alternative proposed SFD [Median (IQR) EEN vs SFD, Patients: 62 (17.8–83.8) vs 23 (6–46.5), *p* = 0.029, Parents: 50.5 (13.3–77) vs 26.5 (3.5–51), *p* = 0.026)] (Fig. 1). There were no significant differences between patients’ and parents’ opinion (Fig. 1) and their ratings were strongly correlated (EEN: *r* = 0.831, SFD: *r* = 0749, both *p* < 0.001).

Participants generally agreed that if they needed to undertake a further EEN course in a future relapse of their disease, they would agree to participate in a clinical trial comparing EEN with an SFD (Patients: *n* = 23; 79%, Parents: *n* = 21; 72%). When we explained further the design of a hypothetical RCT, and reported that the development of a new dietary treatment could decrease medication exposure, these percentages remained equally high (Patients: *n* = 25; 86%, Parents: *n* = 20; 69%) (Table 2).

To further explore these data, we split each of the patient and parent groups into 4 further subgroups based on whether they completed their previous EEN course or not and whether they used an NG tube during treatment or not. This subanalysis revealed that participants who failed treatment generally had a negative attitude to an EEN repeat. In addition, participants who didn’t use an NG tube had a more positive attitude towards the use of an SFD (Table 2).

**Fig. 2:**
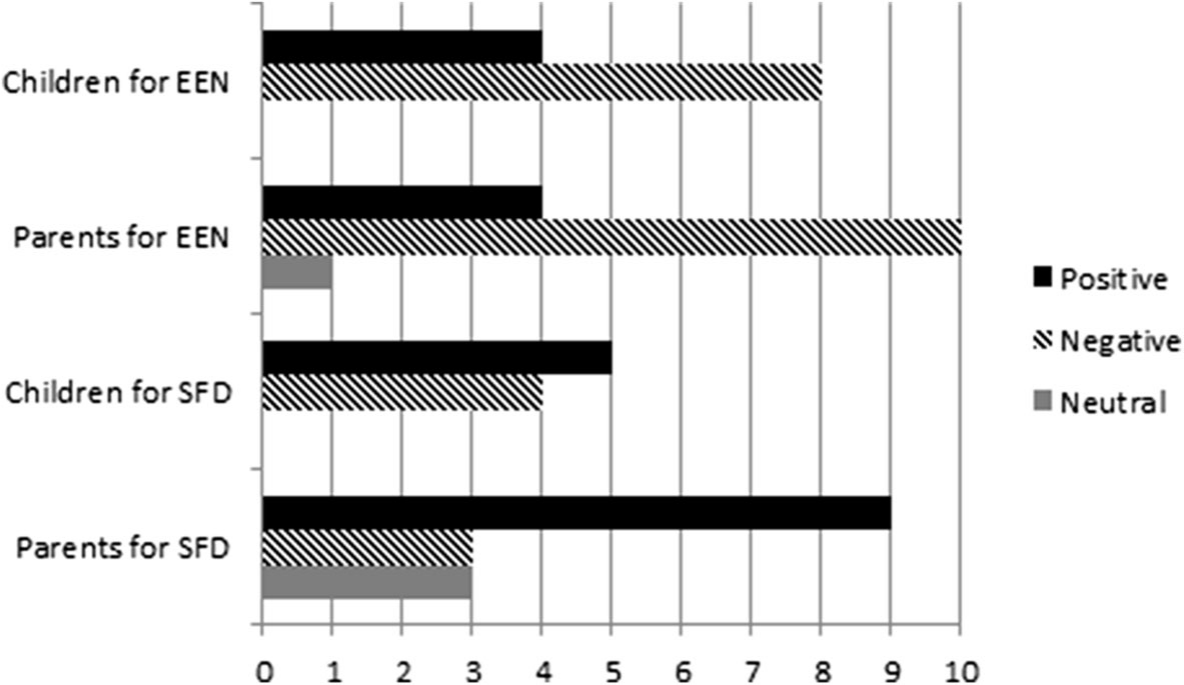
Comments received by Crohn’s disease patients and their parents regarding exclusive enteral nutrition and solid food-based diet. EEN: Exclusive Enteral Nutrition, SFD: Solid Food-based Diet

When these participants were asked to provide any further comments on an open-ended question, we received 51 quotes on EEN and SFD (full quotes list available on request). The majority of the EEN comments were negative [positive vs negative vs neutral; Parents: 4 (27%) vs 10 (67%) vs 1 (7%), Patients: 4 (33%) vs 8 (67%) vs 0 (0%)]. The opposite was observed for the SFD comments [Parents: 9 (60%) vs 3 (20%) vs 3 (20%), Patients: 5 (56%) vs 4 (44%) vs 0 (0%)] (Fig. 2). Selected quotes included: “the liquid-only diet was very isolating at times for my child”; “my child found the liquid diet easy as it was through the tube”; “I think an SFD would be difficult to maintain without temptation”; “I think being on the SFD may make her feel more normal and part of the family”.

Regarding the adult CD patients previously treated with EEN over 1 year, 10 were identified of whom only 3 responded after the reminder letter. The responders’ perceptions towards the use of an SFD were similarly positive to those of CD families but these data are not presented due to the very low response rate.

## Discussion

This survey delivers important insights on the EEN experience for families of children with CD and explores the acceptability of an alternative hypothetical SFD. The large majority of our participants would be happy to repeat an EEN course during a further relapse of their disease. This indicates that both patients and their carers recognise the efficacy of EEN in CD management. The modern use of more palatable polymeric feeds, and the experience and training of health care professionals involved in administering the treatment are known factors increasing the acceptability of EEN [15, 16].

Despite the positive attitude to the use of EEN, most respondents would preferentially agree to use an alternative SFD. The existing literature, describing patients’ frequent requests for dietary advice and exclusion of certain foods to prevent future relapses, is supportive of the idea that an SFD would be well-received [17, 18].

CD patient perceptions were not different and strongly correlated to those of their parents. This strong agreement between parents and their chronically ill children has been reported before [19]; however, there are strong arguments that both opinions are of vital importance and should be sought jointly [20].

The present survey is not without its limitations. Our participants were asked to compare two different dietary treatments, having experienced only the EEN before. They therefore had to provide a hypothetical view on an SFD, based on a provided exclusion diet template. Additionally, only paediatric data are presented in the current publication due to the poor response rate (30%) in the adult CD patients approached for the reasons of this survey. EEN use in adult CD patients is not a standard practice due to limited evidence of its efficacy in historical datasets. Poor compliance mainly explained by palatability issues is reported as the main reason for this [4]. The low response rate among these patients could be explained by their disease status, as according to the current guidelines EEN use in adults is biased towards patients with drug resistance or used as an adjunctive therapy [21, 22]. Another potential source of bias is the EEN completion rate within the 12 patients (29% non-respondents) who did not return their questionnaires. Lack of disease response on EEN course may differ between respondents and non-respondents and this was not specifically examined in this cohort. The EEN completion rate within the 29 families included in our results (79%) however is in broad agreement with previously published rates from the same centre (75%), suggesting a similar group [14].

## Conclusions

In conclusion, surveyed CD patients and their parents are generally happy to repeat a course of EEN if needed, though unsurprisingly this enthusiasm falls with previous EEN failure. Additionally, CD families surveyed are supportive of the development and study of solid food-based dietary treatments. This fits nicely with a well-described desire for dietary modifications amongst the IBD patient community and lends support towards developing a new paradigm of CD dietary therapy, based on the success of EEN.

## Additional files

Additional file 1: Cover letter accompanying the questionnaire forms sent to families of paediatric Crohn’s disease patients. (PDF 214 kb)
Additional file 2: Survey on Dietary Treatment for Crohn’s Disease Child/Young Person Version. (PDF 243 kb)
Additional file 3: Survey on Dietary Treatment for Crohn’s Disease Adult/Carer Version. (PDF 241 kb)
Additional file 4: Raw data analysed during this study. (XLSX 15 kb)

## Abbreviations

CD: Crohn’s disease
EEN: Exclusive enteral nutrition
IBD: Inflammatory bowel disease
NG: Nasogastric
SFDs: Solid food-based diets
