# Supplementary material for: Dietary treatment of Crohn’s disease: perceptions of families with children treated by exclusive enteral nutrition, a questionnaire survey
Source: BMC Gastroenterol. 2017 Jan 19;17:14. doi: 10.1186/s12876-016-0564-7 (PMC5247812; doi:10.1186/s12876-016-0564-7)
Supplement: Additional file 2: — Survey on Dietary Treatment for Crohn’s Disease Child/Young Person Version. (PDF 243 kb) [file 12876_2016_564_MOESM2_ESM.pdf]

Survey on Dietary Treatment for Crohn's Disease
Child/Young Person Version

You were given a special liquid-only diet during 2015. This was used to treat your bowel when you were diagnosed with Crohn's disease or when you were having a flare-up. This was most likely to be a feed called 'Modulen IBD'. We want to know how it went and what you would think of a new study looking to compare this liquid-only diet with a new solid food diet for Crohn's disease.

(Q1) Do you have inflammatory bowel disease?

Yes ☐

No ☐

(Q2) How old are you?

Age: ______years

(Q3) Are you a: Boy ☐ Girl ☐

(Q4) Did you receive a liquid-only diet for a number of weeks?

Yes ☐

No ☐

(Q5) Did you manage to finish 8 weeks of liquid-only diet?

Yes ☐

No ☐

If Yes, please move on to Q8, otherwise please complete Q6.

(Q6) Why did you not manage to complete the course?

We have left this question open for you to answer. Please provide as much detail as possible. It would be helpful for us to know how many weeks you managed to finish.

____

<u>Survey on Dietary Treatment for Crohn's Disease</u>
<u>Child/Young Person Version</u>

For Q7 and Q13 please make a mark on the line under the question to indicate the best answer.

(Q7) How easy was it for you to take the liquid-only diet?

No Problem	________________________________	Impossible

(Q8) Did you need a nasogastric tube for the liquid-only diet?

Yes- feeding pump used ☐

Yes- syringe "bolus" used ☐

No ☐

(Q9) Was this your first course of liquid-only diet?

Yes ☐

No ☐

(Q10) If the answer to Q9 was No, how many previous courses have you had?

____

(Q11) If you had a further flare-up of Crohn's disease, do you think you could complete another course of liquid-only diet?

Yes ☐

No ☐

We are currently designing a new diet based on the milks used for liquid-only diet, but using solid food rather than entirely liquid. We have attached an example on Page 5. The diet we propose has not yet been tested in patients with Crohn's disease, so would first need a scientific study comparing it directly to the liquid-only diet.

(Q12) Do you think a solid food based diet would be better than the liquid diet?

Yes ☐

No ☐

(Q13) How easy do you think it would be to take a solid food diet like the one proposed on Page 5?

No Problem	————————————————	Impossible

(Q14) Do you think it would be easier to complete 8 weeks on a solid food diet, like shown on Page 5, or the liquid-only diet?

Solid food ☐

Liquid-only ☐

(Q15) Would you be interested in participating in a study of a solid food diet, like the one on Page 5, compared with liquid-only diet?

Yes ☐

No ☐

The best way to conduct a study comparing a new treatment and an old one, that we know works well, is by a randomised controlled trial. In a randomised controlled trial patients who we think would benefit from the liquid-only diet, and who agree to take part in the study, would be split evenly (50:50) between it and the new treatment. The choice would be made randomly, for example by tossing a coin, to make sure both groups were similar.

(Q16) Would you agree to take part in such a study if doctors felt you needed to repeat the liquid-only diet?

Yes ☐

No ☐

Survey on Dietary Treatment for Crohn's Disease
Child/Young Person Version

(Q17) Would you take the solid food diet (outlined on Page 5) beyond 8 weeks if it was effective and meant less medication?

Yes ☐

No ☐

(Q18) Do you have any other comments on the liquid-only diet or the solid food diet proposed?

____

____

____

____

____

Thank you for taking the time to complete this survey. We asked very similar questions to your parent, but we want to see whether children and parents have different views on liquid diet in Crohn's disease. We'd appreciate both questionnaires being completed and returned together.

The IBD Team at Yorkhill.

Please return the surveys together in the provided stamped addressed envelope.

Example Diet

Please note this is **NOT** the actual diet we are proposing. It is very important that the solid food diet is tested rigorously in children with Crohn's disease in a scientific manner before we start using it, so we have designed an example based on the kind of restriction to normal diet that would be needed. The diet proposed on this sheet will not treat or help Crohn's disease and **should not be followed**.

Breakfast
Certain types of dairy products
Certain types of breakfast cereals
Certain types of juices

Morning Snack
Certain types of juices
Certain types of bread, dairy and meat products

Lunch
Certain types of bread and starchy foods
Certain types of dairy and meat products
Certain types of vegetables
Certain types of cooking and salad oils

Afternoon Snack
Certain types of dairy products
Selected types of fruits and vegetables

Dinner
Certain types of bread and starchy foods
Certain types of dairy and meat products
Certain type of vegetables
Certain types of cooking and salad oils
