# Supplementary material for: Dietary treatment of Crohn’s disease: perceptions of families with children treated by exclusive enteral nutrition, a questionnaire survey
Source: BMC Gastroenterol. 2017 Jan 19;17:14. doi: 10.1186/s12876-016-0564-7 (PMC5247812; doi:10.1186/s12876-016-0564-7)
Supplement: Additional file 3: — Survey on Dietary Treatment for Crohn’s Disease Adult/Carer Version. (PDF 241 kb) [file 12876_2016_564_MOESM3_ESM.pdf]

Survey on Dietary Treatment for Crohn's Disease
Adult/Carer Version

Your child had a special liquid-only diet under the care of the Yorkhill IBD team during 2015. This was used to treat their Crohn's disease either at the time they were diagnosed or when their disease flared up. This was most likely to be a feed called 'Modulen IBD' but for younger children it may have been a feed with a different name. We want to know how it went and what you would think of a new study looking to compare this liquid-only diet with a new solid food diet for Crohn's disease.

(Q1) Are you the parent of a child with Crohn's disease?

Yes ☐

No ☐

(Q2) Do either of the parents of your child have inflammatory bowel disease?

Yes ☐

No ☐

(Q3) About your child:

Age: ______years, ______months (example: 12 years, 8 months)

(Q4) Is your child: Male ☐ Female ☐

(Q5) Did your child receive a liquid-only diet for a number of weeks?

Yes ☐

No ☐

(Q6) Did your child complete 8 weeks of liquid-only diet?

Yes ☐

No ☐

If Yes, please move on to Q8, otherwise please complete Q7.

(Q7) Why did your child not complete the course?

We have left this question open for you to answer. Please provide as much detail as possible. It would be helpful if you could also tell us how many weeks were completed.

____

For Q8 and Q14 please make a mark on the line under the question to indicate the best answer.

(Q8) How easy was it for your child to take the liquid-only diet?

No Problem ———————————————————— Impossible

(Q9) Did your child require a nasogastric tube for the liquid-only diet?

Yes- feeding pump used ☐

Yes- syringe "bolus" used ☐

No ☐

(Q10) Was this your child's first course of liquid-only diet?

Yes ☐

No ☐

(Q11) If the answer to Q10 was No, how many previous courses have they had?

____

(Q12) If your child had a further flare of Crohn's disease, do you think they could complete another course of liquid-only diet?

Yes ☐

No ☐

We are currently designing a new diet based on the milks used for liquid-only diet, but using solid food rather than entirely liquid. We have attached an example on Page 5. The diet we propose has not yet been tested in patients with Crohn's disease, so would first need a scientific study comparing it directly to the liquid-only diet.

(Q13) Do you think such a solid food based diet would be better than the liquid diet?

Yes ☐

No ☐

(Q14) How easy do you think it would be for your child to take a solid food diet like the one proposed on Page 5?

No Problem	———————————————	Impossible

(Q15) Do you think it would be easier to complete 8 weeks on a solid food diet, like shown on Page 5, or the liquid-only diet?

Solid food ☐

Liquid-only ☐

(Q16) Would you be interested in participating in a study of a solid food diet, like the one on Page 5, compared with liquid-only diet?

Yes ☐

No ☐

The best way to conduct a study comparing a new treatment and an old one, that we know works well, is by a randomised controlled trial. In a randomised controlled trial patients who we think would benefit from the liquid-only diet, and who agree to take part in the study, would be split evenly (50:50) between it and the new treatment. The choice would be made randomly, for example by tossing a coin, to make sure both groups were similar.

(Q17) Would you be happy for your child to participate in such a study if doctors felt they needed a repeat of their liquid-only diet for Crohn's disease?

Yes ☐

No ☐

<u>Survey on Dietary Treatment for Crohn's Disease</u>
<u>Adult/Carer Version</u>

(Q18) Do you think your child would take the solid food diet (outlined on Page 5) beyond 8 weeks if it was effective in treating their Crohn's disease and meant less medications?

Yes ☐

No ☐

(Q19) Do you have any other comments on your experience of the liquid-only diet or the solid food diet proposed?

____

____

____

____

____

Thank you for taking the time to complete this survey. We realise the questions being asked of you and your child are very similar, but we want to see whether children and parents have different views on liquid diet in Crohn's disease. We would appreciate both questionnaires being completed and returned together.

The IBD Team at Yorkhill.

Please return the surveys together in the provided stamped addressed envelope.

<u>Example Diet</u>

Please note this is **NOT** the actual diet we are proposing. It is very important that the solid food diet is tested rigorously in children with Crohn's disease in a scientific manner before we start using it, so we have designed an example based on the kind of restriction to normal diet that would be needed. The diet proposed on this sheet will not treat or help Crohn's disease and **should not be followed**.

Breakfast
Certain types of dairy products
Certain types of breakfast cereals
Certain types of juices

Morning Snack
Certain types of juices
Certain types of bread, dairy and meat products

Lunch
Certain types of bread and starchy foods
Certain types of dairy and meat products
Certain types of vegetables
Certain types of cooking and salad oils

Afternoon Snack
Certain types of dairy products
Selected types of fruits and vegetables

Dinner
Certain types of bread and starchy foods
Certain types of dairy and meat products
Certain type of vegetables
Certain types of cooking and salad oils
