# Supplementary material for: Dietary treatment of Crohn’s disease: perceptions of families with children treated by exclusive enteral nutrition, a questionnaire survey
Source: BMC Gastroenterol. 2017 Jan 19;17:14. doi: 10.1186/s12876-016-0564-7 (PMC5247812; doi:10.1186/s12876-016-0564-7)
Supplement: Additional file 1: — Cover letter accompanying the questionnaire forms sent to families of paediatric Crohn’s disease patients. (PDF 214 kb) [file 12876_2016_564_MOESM1_ESM.pdf]

NHS Greater Glasgow and Clyde

IBD Team

Ward 5B

Royal Hospital for Sick Children

Dalnair Street

Glasgow

G3 8SJ

Dear Parent,

We are surveying the families of all our patients who have received liquid diet treatment for Crohn's disease in the last year. We are doing this for two reasons: to find out how it went and how successful it was; and to look at how people would feel about a possible solid food alternative diet we are trying to design.

We have included two copies of a questionnaire as well as a stamped addressed envelope. One questionnaire is for a parent/carer and the other for the child/young person to complete **themselves.** Both are very similar, but we are interested in whether children and their families have similar or different views on liquid diet for Crohn's disease.

We would be very grateful if you could have both questionnaires completed and then return them to us in the envelope provided. If you have any additional comments, please make them at the end of the survey or on the blank reverse of the last page. If you have any questions about the survey, please feel free to contact the IBD team on: #### ### ####.

Thanks for taking the time to read and respond to this survey. We would be extremely grateful if replies could be submitted by 1st May.

Best wishes,

Dr Richard Hansen

Dr Richard Russell

On behalf of the IBD Team at Yorkhill
